# Study of myelin structure changes during the nerve fibers demyelination

**DOI:** 10.1371/journal.pone.0185170

**Published:** 2017-09-21

**Authors:** Natalia N. Rodionova, Elvin S. Allakhverdiev, Georgy V. Maksimov

**Affiliations:** 1 Faculty of Biology, M.V. Lomonosov Moscow State University, Moscow, Russia; 2 Faculty of Fundamental Medicine, M.V. Lomonosov Moscow State University, Moscow, Russia; University of Hyderabad School of Life Sciences, INDIA

## Abstract

Raman, NMR and EPR spectroscopy and electrophysiology methods were used to investigate the excitability and the packaging of myelin lipid layers and its viscosity during nerve exposure to pronase E. It was established that during exposure of nerve to pronase E the action potential (AP) conduction velocity and the Schwann cell (SC) (or myelin) water ordering increases, but the nerve myelin refractive index and internode incisions numbers decrease. This effect included two periods–short- and long-time period, probably, because the first one depends on SC protein changes and the second one–on the nerve fiber internode demyelination. It was concluded that high electrical resistance of myelin, which is important for a series of AP conduction velocity, not only depends on nerve fiber diameter and the myelin lipid composition, but also on the regularity of myelin lipid fatty acids and myelin lipid layer packing during the axoglial interaction.

## Introduction

Repetitive excitation of a nerve fiber is known to result in accumulation of extracellular K^**+**^, especially between the axon and the myelin [1,2]. Uptake of K^**+**^ leads to Schwann cell (SC) swelling and myelin restructuring that impacts its electrical properties. In some pathology, such as nerve ischemia, demyelination, and disruptions of junctions between the axon and glial cell (CNS), which fails to cope with the K^**+**^ uptake, fluxing out through the open K^**+**^-channels of the internode axolemma. Because of this failure, K^**+**^ accumulates in the confined extracellular space between the axon and the sheath (internode space), and to a lesser extent around the node of Ranvier (NR) and outside of the fiber. The nodal membrane is enriched with sodium channels but poor in fast potassium channels, whereas the intermodal membrane is enriched with fast potassium channels but has relatively few sodium channels. The high density of sodium channels in the node needs little or no comment because saltatory conduction requires a large inward current at the node. The frog internode contained a very low density of sodium and potassium channels compared with the density of both channels types in the nodal membrane. The potassium conductance’s, particularly G_Ks_, also control the nodal and intermodal resting potentials. Analysis of K^+^ -currents in rat nerve fibers after demyelination during the perfusion with pronase or lysolecitin suggests the existence of two fast and one slow potassium channels populations, similar in the nodal membrane of frog nerve fibers [3,4].

Pronase E is one of the proteolytic enzymes that is often used for studying of the nerve demyelination [5,6]. Although several effects of pronase E on the physiology of non-neuronal tissues have been described, the effects of these enzymes on myelin nerve fiber have received little attention. It was examined that the effects of bath-applied pronase E are caused by broadening of neuron AP, that exhibits a shoulder on the AP repolarization phase. This effect was accompanied by membrane depolarization and decreased interval between AP. Some, but not all, effects of pronase E were reversible: the membrane potential and AP interval changes were both reversed after ∼1 h of washing, unlike the AP duration (after 90 min). Pronase E neither changed the inactivation rate nor caused a shift in the current-voltage relationship of the voltage-activated Ca^2+^ currents in neurons [7]. After enzymatic demyelination with pronase E, fast K^+^-currents of large amplitude appeared in rat and frog myelinated nerve fibers (K_f1_ and K_f2_ channels) [8,9]. Perfusion of internal axons with pronase selectively disturbs inactivation of the Na^**+**^-conductance. Tetrodotoxin does not protect the inactivation gates from pronase, nor maintains inactivation of the Na^**+**^ -channels during nerve exposure to pronase [10].

Recently, the myelin-dependent effects were shown on nerve fibers using Raman spectroscopy [11–15]. Carotenoids in myelin can possibly mediate the potential-induced myelin reorganization, as well as regulate the interaction of external signaling molecules with the membrane. It was shown that both lipid and carotenoid molecules are sensitive to the extracellular ATP and acetylcholine, and thus are possible mediators of the myelin membrane restructuring [13–15]. The aim of current investigation was to study the conformation of myelin phospholipid fatty acids and carotenoid during demyelination.

## Materials and methods

### Nerve fibers preparation

All experiments were performed on myelinated nerve fibers of frog *Rana temporaria*. All experiments on animals (we used 70 animals aged twelve months from the vivarium of the Faculty of Biology, M.V. Lomonosov Moscow State University) were carried out in accordance with the animal care regulations of the M.V. Lomonosov Moscow State University. The protocol was approved by the Bioethics Committee of the Faculty of Biology, M.V. Lomonosov Moscow State University. Prior to experiments, male frogs were maintained in containers with some water at 5°C without feeding. Animals were anesthetized using solution of propofol (50 mg/kg) administered intracelomically, which ensured the deep anesthesia condition prior to euthanasia. Anesthetized animals were decapitated with the following pithing of the brain.

Nerves were dissected and then incubated in Ringer buffer solution (RBS) consisting of 100 mM NaCl, 2 mM KCl, 1.08 mM CaCl_2_, and 10 mM HEPES (pH 7.4) for at least 30 min. Single nerve fibers were obtained by splitting each nerve. Experiments were carried out at room temperature (20–22°C). Nerve fibers were placed on a glass bottom of a FluoroDish (World Precision Instruments, USA) filled with RBS and then covered by a glass coverslip [16].

### Phase imaging

Nerve imaging was done with an automated interference profilometer, developed at VNIIOFI (Moscow, Russia) and based on the MII-4 Linnik interferometer (LOMO, St-Petersburg, Russia) [17]. The device allows to obtain the images of transparent and semitransparent objects in the reflected light as well as interference images. The x30 objective with 0.65 numerical aperture was used. The registration frame size was 196x145 μm. Images were acquired with a WinPhast software. Nerve phase image reconstruction from interference images was achieved by a phase-stepping technique. The black and white 12 bit CCD camera, 415U (Videoscan, Russia), with a 1/2 inch sensor (6.5*4.83 mm) and resolution of 782x582 pixels was used to capture images. For each measurement ten interference images were obtained. The acquisition time of an image was limited by the exposure time of the camera (normally about 20 ms). The total time of acquisition and phase image reconstruction was about 10 s. Samples were illuminated with a 650 nm 5 mW diode laser (the power per cell was about 2.3 mW). Vertical resolution of the device was λ/200 nm and the lateral resolution was 0.5 μm.

### Electron paramagnetic resonance

To evaluate the nerve myelin viscosity, the electron paramagnetic resonance (EPR) was used. The spin-spin labeled derivative of stearic acid (DS-16) (Sigma) was used as a probe. It is known, that nitroxyl radical probe DS-16 is localized at a distance not less than 2.2 nm from the surface of the plasma membrane and it allows recording the membrane viscosity at the center of the hydrophobic myelin membrane layer. To evaluate the membrane viscosity, the correlation time (τ) of nitroxyl radical at the EPR spectra was calculated [18]. In the experiment, nerve was incubated in RBS with DS-16 (10^−4^ M) for 10 minutes (alcohol solution, 3.5 mmol/l), then the nerve was placed in a glass capillary with RBS without DS-16. After that, EPR spectra were recorded with the following parameters: constant magnetic field—3338 G, power 22 mW, with a time constant of 0.1. ESR spectrometer RE-1308 (Russia) and the ESR spectrometer ESR-Analyzer / MMS, Series 01–08 (Germany) were used. Measurements were carried out at 18–20°C.

### Nuclear magnetic resonance (NMR)

To characterize the degree of mobility of the water protons, their nuclei spin-spin relaxation time (T_2_) was determined after the application of a high-frequency pulse [19], using the method of Carr-Purcell-Meiboom-Gill and NMR "spin echo": a constant magnetic field (H_0_) was about 0.4 T; high frequency magnetic field (H_1_) was 17 MHz, the burst flow rate reached 9 times per second, the accumulation of 100 and the measurement accuracy was better than 7%. To eliminate the influence of the water protons from incubation medium on the signal during T_2_ registration, nerve was placed in RBS with D_2_O. The value of T_2_ was 800–1000 ms, which is typical for the nuclei of water protons [19].

### Raman spectroscopy

For Raman spectroscopy study of nerve fibers, the confocal Raman spectrometer NTEGRA Spectra (NT-MDT, Russia) with a 532 nm and 780 nm laser excitation was used. Raman spectra were recorded by focusing the laser beam on the nerve fiber myelin surface through the ×40 objective with NA 0.6 (Olympus, Japan). Laser power was set to 0.8 mW and integration time for each spectrum was 50–60 s. Spectrometer has a grating with 600 lines/mm and entrance slit set to 100 μm. Raman scattered light was collected in a backscattering mode and detected with a CCD camera (1024 x 256 pixels, -50°C). Raman spectra baseline subtraction and calculation of Raman peak intensities were made using OriginPro 2015 software (OriginLab Corporation, USA) [20, 21].

### Action potential (AP)

AP was registered via bipolar extracellular potential method. For nerve stimulation, rectangular pulses of super threshold voltage were used with 0.3 ms duration, amplitude of 0.8–1.0 V, and 100 Hz repetition frequency of stimulating pulse -. Using LGraph program, the AP amplitude and velocity of propagation were calculated.

Statistical analysis was carried out using the Kruskal-Wallis test. Statistical significance level was set at 1% (p <0,001).

## Results and discussion

### Study of the nerve excitability and morphology changes during pronase E demyelination

**Fig 1:**
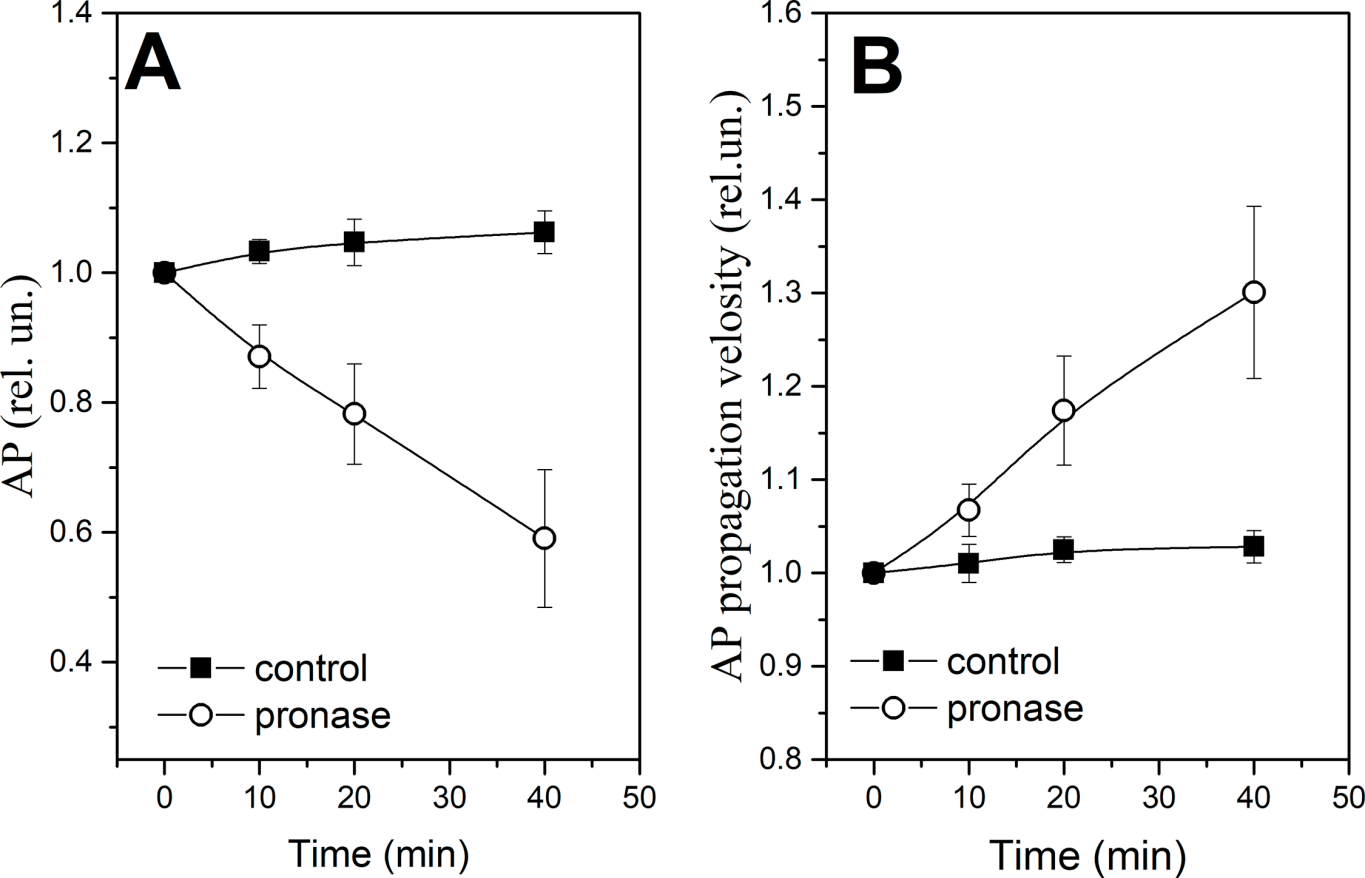
**The amplitude (A) and the velocity of AP propagation (B) changes during nerve exposure to pronase E (0,2%).** Significant difference (p <0.05).

**Fig 2:**
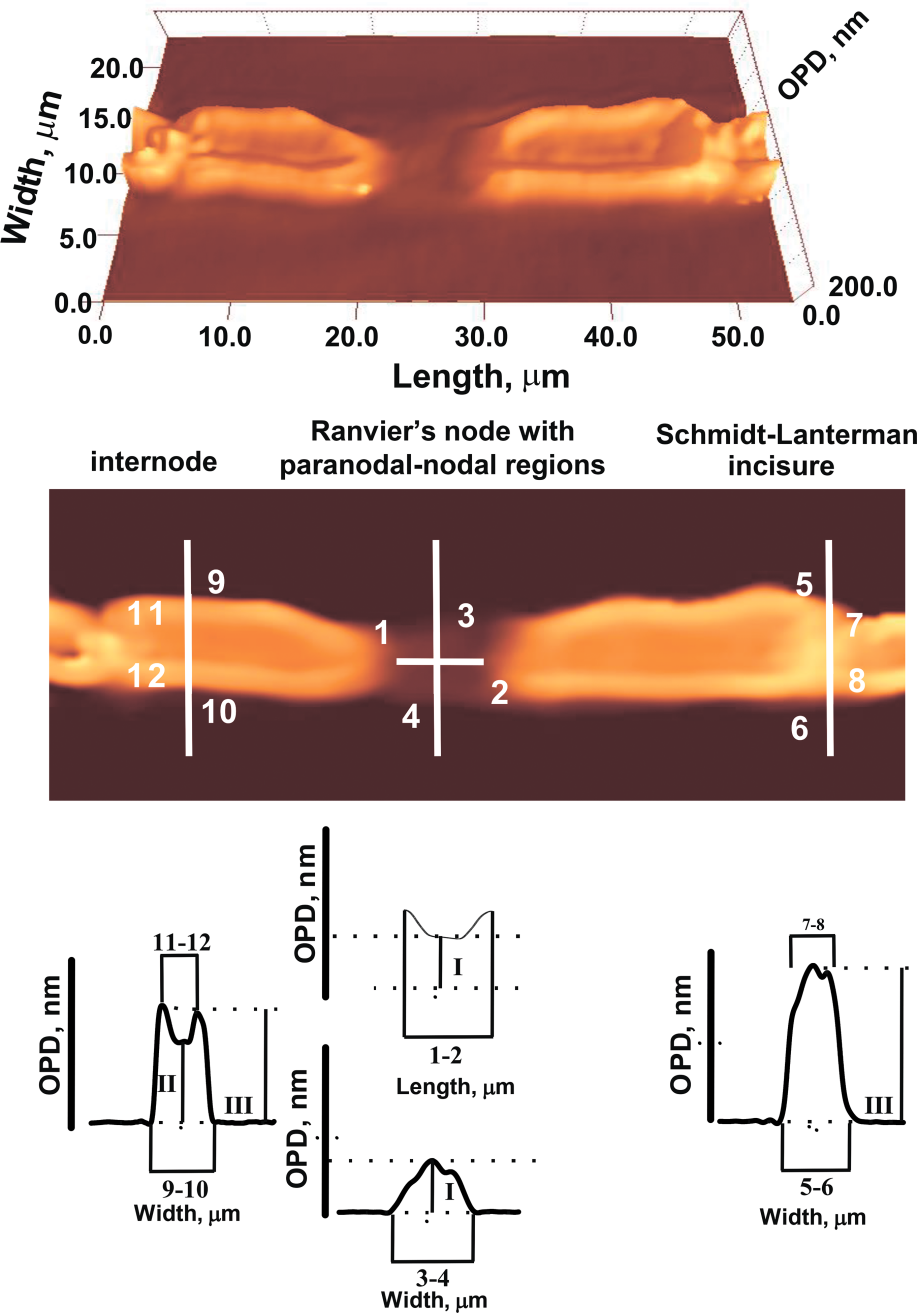
The phase image parameters of myelin nerve fiber (1–12). (1–2)—the paranodal areas with Ranvier’s node length; (3–4)—Ranvier’s node diameter; (5–6)–diameter of nerve fiber internodal region (in incisures area); (7–8)—diameter of axon in incisures area; (9–10)—diameter of nerve fiber in internodal region; (1–12)—diameter of axon in internodal region; I—the OPD in Ranvier’s node; II—the OPD in the nerve fiber center; III- OPD in the nerve fiber boundary.

**Fig 3:**
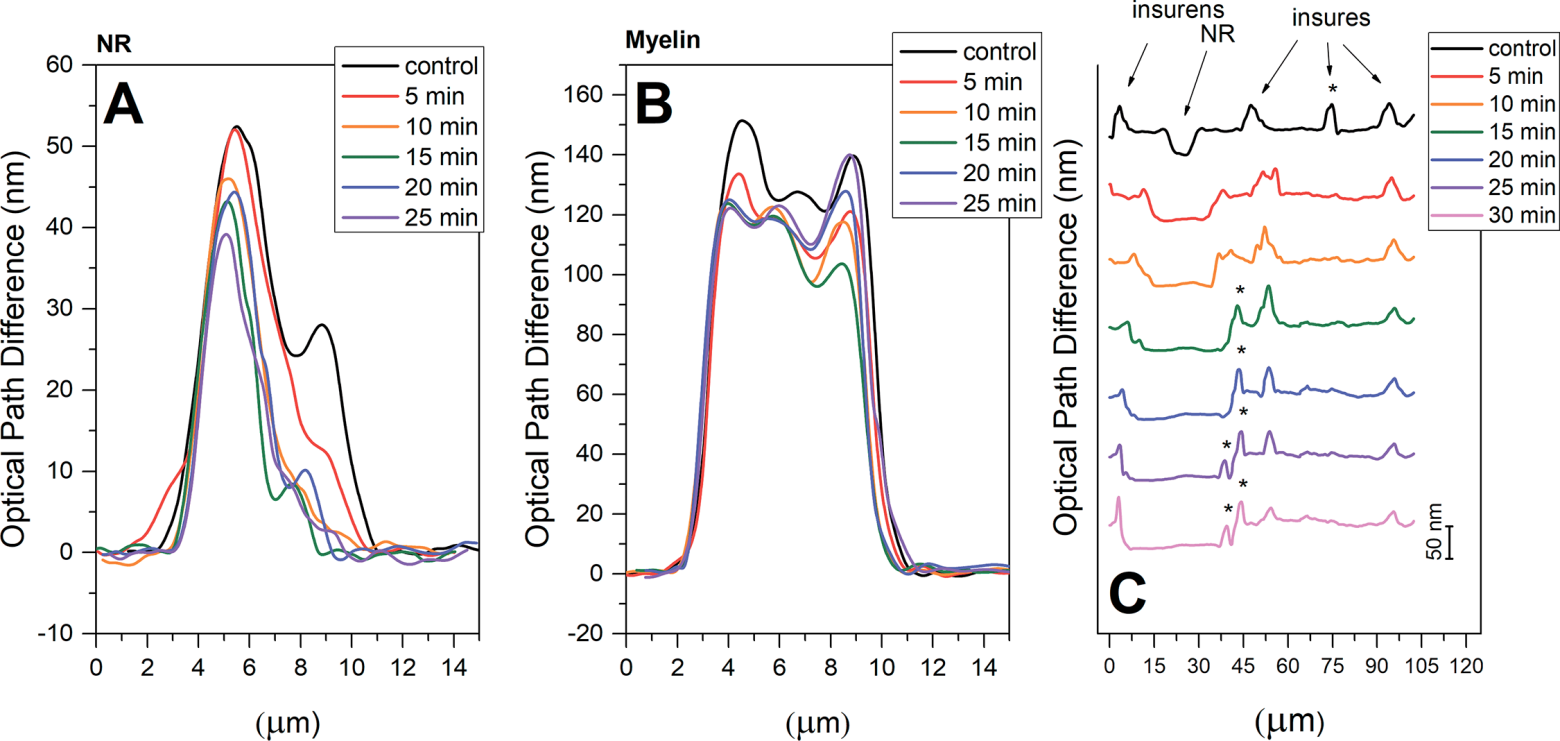
The dynamics of OPD profile changes along the scan-line (μM) through NR. (A); in internode region of the myelin fiber (B); the perpendicular to the normal nerve fiber and along the nerve fiber (C), during exposure of the nerve to pronase E.

We have studied the effects of the proteolytic enzyme pronase E on the nerve excitation (AP amplitude and AP conduction velocity). It was found that during 10 min incubation of the nerve in RBS with pronase E the AP amplitude decreased and the AP conduction velocity increased (Fig 1). Indeed, the decrease of the AP amplitude might be associated with both an increase in the excitation threshold during the activation of additional internode K^+^-channels and the inactivation and redistribution of the NR sodium and potassium channels [4,5]. Using LIM, we studied the morphology changes in different regions of nerve fibers: axon diameter and length in Ranvier’s node (NR), nerve fiber and axon optical path difference (OPD) (Fig 2). It was found, that during nerve exposure to pronase the NR length increases and the numbers of the incisions decrease (Fig 3). Probably, this changes both increase myelin lipid layer packing (no only ordering of lipid fatty acids) and its electrical resistance and the number of NR channels, which leads to an increase in the AP conduction velocity. In frog nerve, the entry of sodium ions is responsible for the inward fast depolarization that underlies the action potential. The internode myelin has a capacitance two to three orders of magnitude greater than that of the node, but also contains an ionic channel, which plays an important role in complex interaction of node and internode. It might be connected not only with the myelin lipid layer packing and increase the length in Ranvier’s node (distribution of the sodium channels), but also in the reversible changes either the shape or permeability of SC membrane or the axolemma structure. In the latter case, the changes in interaction of myelin proteins with axolemma lead to greater participation of the axolemma ion channels (previously closed myelin) in the AP generation. It is known that the axon Na^+^-channels, K^+^ channels and Nr-CAM and NF 186 protein are connected to axonal cytoskeleton by ankyrin G spectrin [22]. The importance of axoglial interaction in differentiation of axolemma, especially regarding sodium channel distribution (homogeneously distributed sodium channels or SC formation of sodium channels aggregates) was found earlier [23].

**Fig 4:**
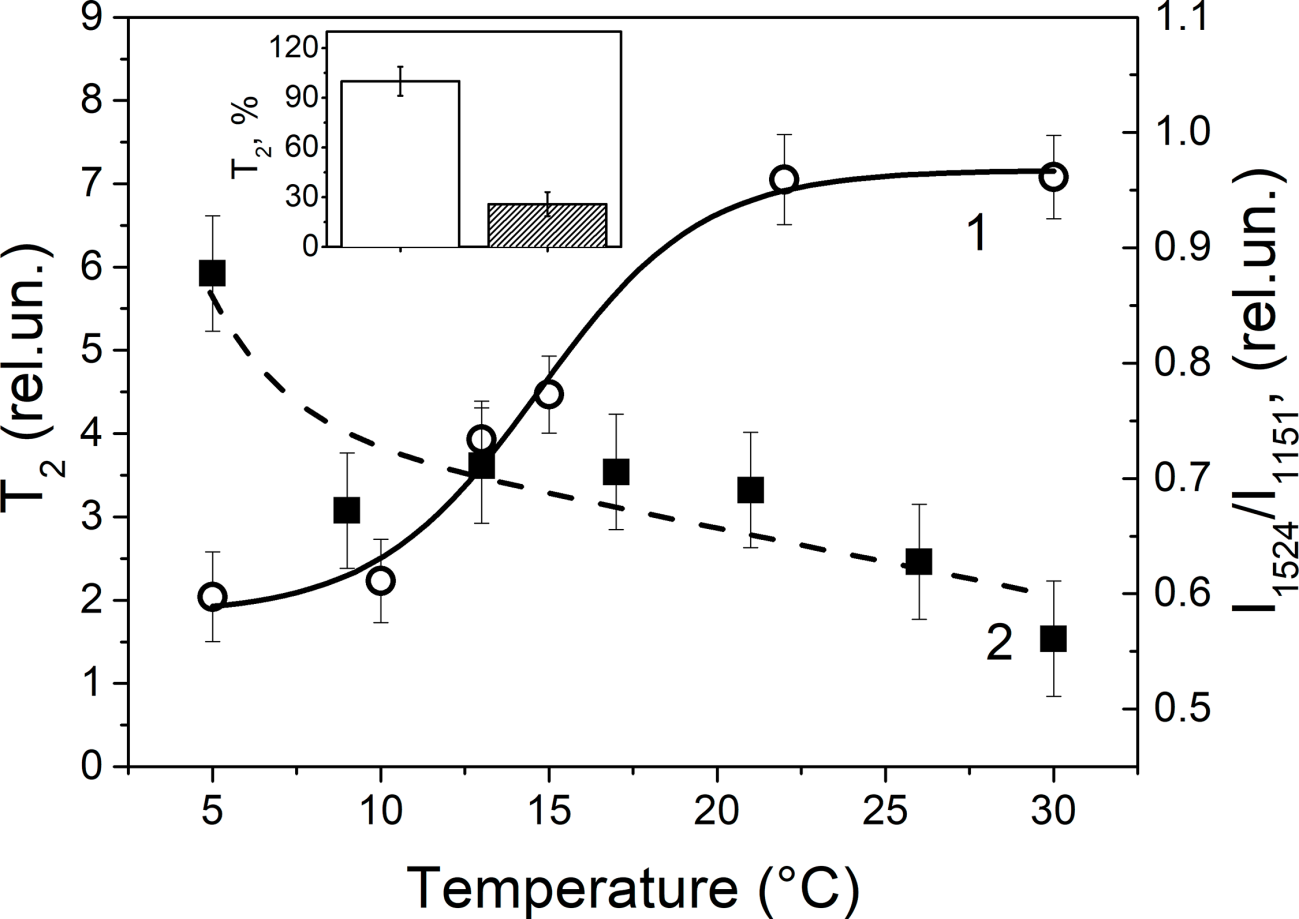
**The temperature dependence of nerve fibers**: on water T_2_ (1) and on the intensity ratio I_1524_/I_1151_ of carotenoids Raman spectra (2) changes. (Insert) The spin-spin relaxation time of protons (T_2_) during 40 min nerve exposure to pronase E. (control (nerve exposure to D_2_O)—white column; nerve exposure—shaded column). The mean value of T_2_ in the control corresponding to 849 ms is taken as 100%. Significant difference (p <0.05).

Next, using NMR "spin echo", the mobility of water protons directly bound to the proteins and/or lipids of the myelin lipid layer during nerve exposure to pronase E was studied. At few minutes of the nerve exposure to pronase E, the myelin and/or SC cytoplasm water spin-spin relaxation time (T_2_) decreased (Fig 4). Probably, this indicates an increase in the ordering of the water molecules structure in some nerve fiber regions (SC or internode space of the nerve fiber). It might be possible that in this fiber region the proteins (channels) and lipid molecules are organized differently, and the hydrophilic sites with the water bound appear. Apparently, proteolysis and demyelination could increase the exposure of protein charged groups, and it can participate in hydrogen bonding with water molecules. It should be noted that in these experiments nerve fiber was placed in the RBS with D_2_O, so H_2_O was only in the SC (for example, in space between the myelin lipid layers), in axon or between axons and SC. Using NMR "spin echo" and RS we studied the temperature dependency on the nerve myelin lipid fatty acids and the SC water molecules regularity (Fig 4). It was established that the dependence of NMR T_2_ and ratio I_1524_/I_1151_ of nerve carotenoids RS on temperature was antiparallel (Fig 4): upon heating of the nerve fiber the ordering of the myelin lipid fatty acids decreases and myelin bound water increases. It is known that the nerve demyelination was made by using osmotic shock [2]. We cannot exclude the role of the SC cytoplasm water in myelin lipid layer packing regulation during nerve excitation: in this case the myelin lipid fatty acids regularity increases because the proportion of structured water (or water in the SC cytoplasm) in myelin decreases.

Thus, the alteration in the structure of the extracellular sites axolemma (or SC) and myelin proteins during nerve exposure to pronase E changes the nerve fiber morphology, the AP conduction velocity, the nerve myelin lipid viscosity and SC water regularity.

### Study of the nerve myelin lipid viscosity changes during pronase E demyelination

**Fig 5:**
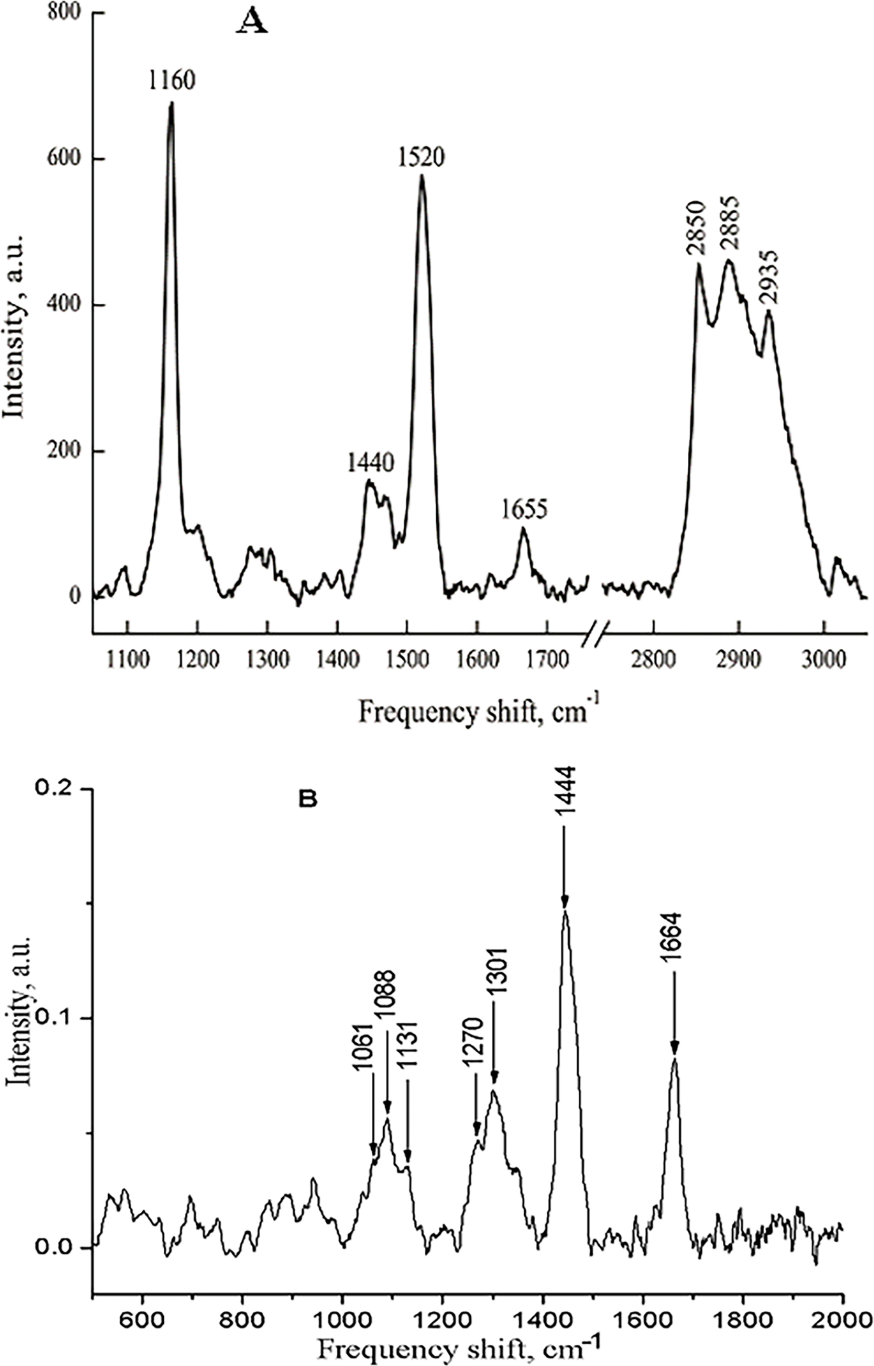
Raman spectra of myelin sheath of sciatic nerve fiber. Raman scattering was excited by 532-nm (A) and 780 nm (B) laser with 0.8 mW power, integration time was 50 s.

In following experiments, the changes in conformation of myelin lipids and carotenoids (the minor membrane component) were studied during the long-lasting nerve fibers exposure with pronase E. As described in detail previously [14,15], the Raman spectrum of nerve fiber myelin could be characterized by the presence of a set of intensive bands, related to phospholipid fatty acids and carotenoids (Fig 5A and 5B). About this, we used another laser excitation wave, which made it possible to reduce the contribution of carotenoid molecules from the RS and to study only the changes in fatty acids of phospholipids. In these experiments, we investigated the changes in regularity of myelin lipid fatty acids using RS lipid scattering (514 and 785 nm laser excitation). It is known that it is possible to test the changes of myelin lipid molecules by means of RS: the relationship between RS band intensities I_1061_/I_1088_ is the proportional ratio of the number of C-C bonds in the trans conformation to the number of C-C bonds in the gauche conformation; I_1270_/I_1301_ is proportional to the number of double bonds of lipid fatty acids; I_1444_/I_1301_ is proportional to the ordering of lipid fatty acids in the membrane. As it was previously shown, carotenoids as the part of the myelin membrane contribute to the bands at 1160 cm^-1^ and 1520 cm^-1^, corresponding to C–C and C = C stretching modes of the polyene chain, respectively [12–15].

**Fig 6:**
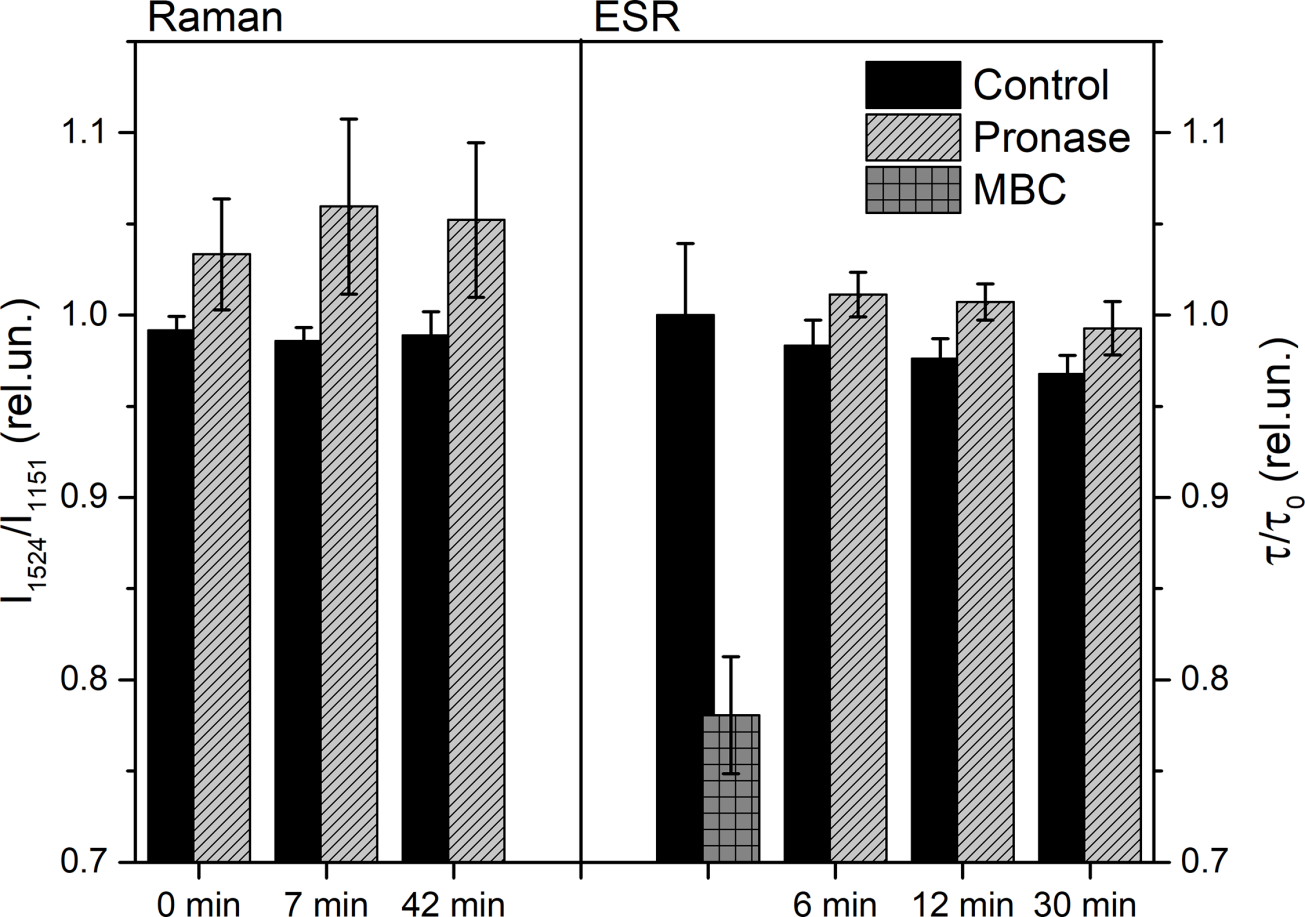
**Changes in the ratio of I**_**1524**_**/I**_**1151**_ **RS bands of carotenoids during nerve exposure to pronase E** (A), and the rotational correlation times (τ) of DS-16 changes during nerve exposure to pronase E and methyl-β-cyclodextrin (10^−2^ M) (B). Significant difference (p <0.05).

To make sure that there are no dynamic changes in values of the band intensities, we recorded Raman spectra of the myelinated nerve fiber kept for 70 min in RBS (control). Indeed, no changes were observed during this experiment. The effect of nerve exposure to pronase E on lipid fatty acids ordering of the myelin bilayers was studed (Fig 6). It was found that pronase E causes changes in RS of the myelin carotenoids: the ratio of the intensities of the I_1524_/I_1151_ bands increases immediately with the maximum up to 7 min. and than returns to the control level. Importantly, there was a second I_1524_/I_1151_ RS maximum (42 min) (Fig 6A).

The correlation between the rotational correlation time (τ) and the ratio of I_1524_/I_1151_ bands of carotenoids RS during the 6–12 min and 30–42 nerve exposure to pronase E (Fig 6B) was found. As we have shown, the nerve myelin carotenoids RS and nerve embedded spin 16-DS probe are correlated during the changes of nerve myelin viscosity [24]. And also, the fact, that during the extraction of nerve myelin cholesterol by methyl-β-cyclodextrin the rotational correlation times (τ) DS-16 decreases, was the evidence of different pronase E mechanism of action. Possibly, during nerve exposure to pronase E, the changes of the myelin viscosity depend on the structure of extracellular site membrane proteins: the changes in proteins initiate increasing regularity of myelin lipid fatty acids. Thus, changes in the structure of the protein sites localized on the extracellular axon surface (or SC) can initiate changes in myelin viscosity, which probably changes the velocity of AP propagation.

**Fig 7:**
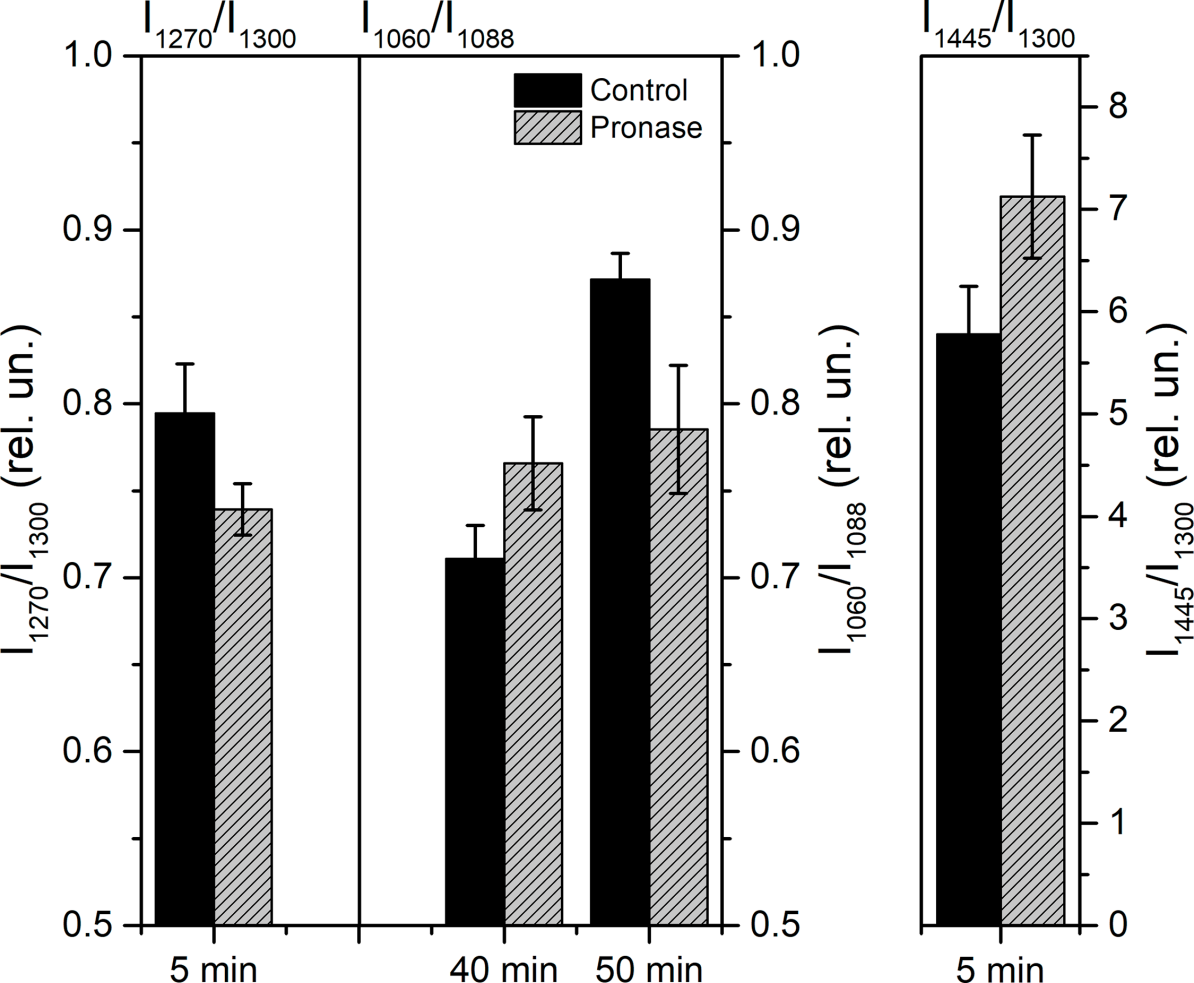
The dynamics of the nerve fatty acids RS peak ratio changes during nerve exposure to pronase E. Raman scattering was excited by 780 nm laser with power of 0.8 mW, registration time was 50 s. Significant difference (p <0.05).

During the nerve exposure to pronase E, the different dynamics of the ordering of the myelin lipid fatty acid molecules was found (Fig 7): (1) during 5–12 min the I_1525_/I_1165_ ratio of the carotenoids, I_1445_/I_1300_ of lipids RS and correlation time (τ) increase, and I_1270_/I_1300_ decreases; (2) during 40–42 min—increasing I_1524_/I_1151_ of carotenoids and I_1060_/I_1088_ of lipid fatty acids RS point to an increase of the nerve membrane ordering and initiation of transformation of C-C bonds in the trans conformation to the these in the gauche conformation. Thus, the extracellular sites of changes of SC membrane or axolemma proteins are accompanied by an ordering of myelin membrane fatty acids during two different periods: short (5–12 min)- and long (40–42 min)- lasting period. It likely depends on both SC protein changes and the nerve fiber internodal region demyelination.

## Conclusions

Thus, it was established that during the nerve exposure to pronase E the AP conduction velocity and the SC (or myelin) water ordering increase, but the nerve myelin refractive index and numbers of internode incisions decrease. The nerve excitation depends on the myelin proteins changes increasing regularity of myelin lipid fatty acids during the axoglial interaction. In the demyelination conditions, the myelin lipid fatty acids and carotenoids exhibit two-time period (short- and long-time). Possibly, the first one is the result of SC membrane protein changes and Ca^2+^ permeability of SC membrane [14, 15], and the second one is the result of the changes in protein conformation and its distribution in the nerve fiber internode (and NR) region. Finally, functional changes in nerve AP conduction, associated with demyelination and dysmyelination, depend on both the myelin abnormalities themselves and the secondary axonal and SC abnormalities. For example, a study of remyelination after lysophosphatidyl choline action present evidence that here too sodium channels aggregates appear at the edges of the myelin sheaths [23]. We might conclude that a high electrical resistance of myelin important for conduction of a series of AP not only depends on the lipid composition, but can also the be the regularity of myelin lipid fatty acids and myelin lipid layer packing during the axoglial interaction.

## Abbreviations

NMR: Nuclear Magnetic Resonance
EPR: Electron Paramagnetic Resonance
AP: Action Potential
SC: Schwann Cell
CNS: Central Nerve System
